# Serum MPO-DNA for Predicting the Risk of Venous Thromboembolism and the Effect of Statins in Patients with Spontaneous Intracerebral Hemorrhage

**DOI:** 10.1055/a-2595-1927

**Published:** 2025-05-09

**Authors:** Xinyan Yan, Wenyan Huang, Yunrong Chen

**Affiliations:** 1 Department of Neurology, Hunan Provincial People's Hospital and The First-Affiliated Hospital of Hunan Normal University, Changsha, Hunan, People's Republic of China; 2 Department of Pulmonary and Critical Care Medicine, Hunan Provincial People's Hospital and The First-Affiliated Hospital of Hunan Normal University, Changsha, Hunan, People's Republic of China

**Keywords:** cerebral hemorrhage, neutrophil extracellular traps, MPO-DNA, venous thromboembolism, hydroxymethylglutaryl-CoA reductase inhibitors

## Abstract

**Background:**

Patients with spontaneous intracerebral hemorrhage (ICH) are at high risk of venous thromboembolism (VTE). Recent studies have shown the involvement of neutrophil extracellular traps (NETs) in thrombogenesis.

**Objectives:**

To explore the predictive value of serum MPO-DNA (a NETs surrogate) for VTE and the effect of statins on serum MPO-DNA levels and the VTE incidence in ICH patients.

**Methods:**

This prospective cohort study enrolled 117 ICH patients and 15 healthy controls. Serum MPO-DNA levels were measured via ELISA. The relationship between serum MPO-DNA levels and VTE risk was analyzed. The predictive value of MPO-DNA was evaluated by ROC curves. Effects of statin on NETs and VTE incidence were evaluated.

**Results:**

The median MPO-DNA level in patients with VTE was 0.304 (95% CI: 0.231–0.349), significantly higher than the 0.188 (95% CI: 0.159–0.236) in non-VTE patients. Elevated MPO-DNA levels were associated with an increased VTE risk (OR 7.13, 95% CI 2.58–19.75; *P* < 0.001), and this association persisted after adjustment. The AUC values for MPO-DNA, CRP, and D-dimer were 0.824 (95% CI: 0.719–0.928), 0.618 (95% CI: 0.481–0.754), and 0.786 (95% CI: 0.683–0.888), respectively. Moreover, statin users exhibited reduced MPO-DNA levels (0.174 vs. 0.218; *P* = 0.007), though VTE incidence differences (13.8% vs. 19.3%) lacked statistical significance.

**Conclusion:**

Serum MPO-DNA serves as a sensitive biomarker for VTE prediction in ICH, highlighting NETs as potential therapeutic targets. Statins could attenuate NETosis, but larger trials are required to validate their clinical efficacy and safety in VTE prevention for ICH patients.

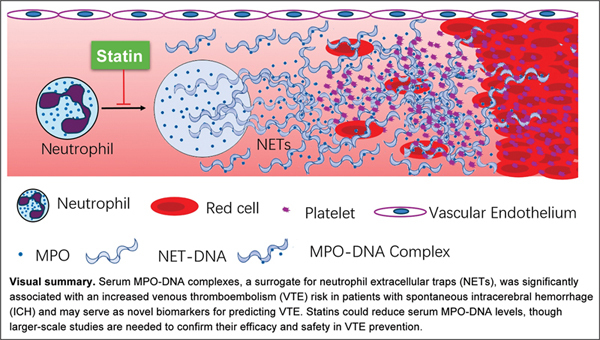

## Introduction

Spontaneous intracerebral hemorrhage (ICH) accounts for about 20% of all strokes, but is the deadliest form of acute stroke, with early-term mortality of about 30 to 40%. 1 2 3 In fact, it is a greater public health threat than conveyed by incidence numbers alone. 4 Patients with ICH are at 4 times higher risk of venous thromboembolism (VTE) than those with ischemic stroke. 5 6 The occurrence of VTE complications was an independent predictor of poor outcome for ICH patients. 7 Patients with VTE had a longer median duration of hospitalization and a higher proportion of intubation. 8 One potential reason for the high incidence of VTE in ICH patients is that clinicians may avoid or delay the use of pharmacological prophylaxis due to concerns about worsening hemorrhage. 4 9 The treatment strategies for ICH and VTE often have conflicting priorities. The focus of VTE treatment is on anticoagulation and preventing thrombus recurrence, whereas the treatment for ICH emphasizes hemostasis and preventing hematoma expansion. Therefore, identifying patients at the highest risk of VTE and finding preventive measures that minimally affect coagulation function are essential areas for investigation.

In recent years, many studies have confirmed that neutrophil extracellular traps (NETs) play a crucial role in VTE formation. NETs, which are composed of decondensed DNA, citrullinated histone H3 (CitH3), and granule proteins such as myeloperoxidase (MPO) and neutrophil elastase (NE), influence VTE formation through several pathways. 10 NETs or their key components have shown potential diagnostic and prognostic value as biomarkers in diseases related to venous thrombosis. What is more, the inhibition of NET formation (NETosis) is sufficient to reduce VTE. Therefore, we hypothesize that levels of serum NETs could serve as a reliable predictor, and that NETosis may represent a therapeutic target for the prevention and treatment of VTE in patients with ICH. To more precisely quantify NETs, it is essential to integrate the measurement of DNA with that of an enzyme specific to neutrophils, such as myeloperoxidase (MPO). Consequently, we assessed serum NET levels by detecting the MPO-DNA complex as previously reported. 11

In this prospective cohort study, we measured the serum MPO-DNA levels in patients with ICH and healthy control, evaluating the predictive value of serum MPO-DNA for VTE. Given that previous studies have shown statins can inhibit NETosis, 12 13 14 we further analyzed the serum MPO-DNA levels and the incidence of VTE in ICH patients receiving statins. Our findings indicated that serum MPO-DNA is an effective predictor for VTE, and that statins can reduce serum MPO-DNA levels in ICH patients.

## Methods

### Study Design

This single-center, prospective cohort study was conducted in hospitalized ICH patients from January 2022 to December 2023. The study was approved by the Clinical Studies Ethical Committee of Hunan Provincial People's Hospital.

### Study Population

Consecutive patients aged 18 years or older with radiographically confirmed ICH admitted to the neurological intensive care unit of Hunan Provincial People's Hospital were included. Patients were screened within 24 hours of onset. Exclusions comprised: (1) pre-admission VTE and (2) hospitalization duration <3 days. In the study, 15 healthy volunteers, whose demographic findings were comparable to the patient group, were included as a control group.

### Data Collection

Upon admission, the following information was recorded on the study form for each patient: demographic characteristics, comorbidities and complications, Glasgow Coma Scale (GCS) scores, radiological findings, and routine laboratory results (including blood counts, serum biochemistry, D-dimer levels, C-reactive protein [CRP], and procalcitonin). Additionally, the treatments and outcomes of patients were recorded. The assessment of VTE risk was conducted using the Caprini scoring system, with risk categorized as low (score 0–1), moderate (score 2), high (score 3–4), and highest (score ≥5).

### Examination of VTE

Patients were assessed for proximal or distal DVT using color Doppler ultrasonography between days 7 and 10 of hospitalization. PE screening was initiated in patients presenting with symptoms such as chest pain, tachypnea, or hypoxemia, and PE was confirmed through pulmonary computed tomography angiography.

### Measurement of Serum MPO-DNA

The serum samples were collected from patients at the time of study entry and were stored at −80°C. Serum MPO-DNA complex levels were measured using a previously described capture ELISA method with slight modifications. 15 To capture the antibody, 96-well microtiter plates coated with anti-MPO polyclonal antibody (1:1000, Invitrogen, **PA5-16672** ) were used, incubated overnight at 4°C and washed 4 times with 0.05% PBS-Tween-20. The plates were then blocked with 1% BSA, and washed 3 times. Next, samples were added together with peroxidase-labelled anti-DNA monoclonal antibody (component no. 2 of the Cell Death Detection ELISA kit, Roche, 11774425001) and incubated at room temperature for 2 h, followed by washing with PBS 3 times. The peroxidase substrate (Roche, 11774425001) was added. After incubation at 37°C for 40 min, the optical density was measured at 405 nm using a microplate reader.

### Statistical Analysis

Continuous variables were expressed as medians with interquartile ranges (IQR) and were compared using the Mann-Whitney U test. Categorical variables were expressed as numbers and percentages (%) and were analyzed using the Chi-square test. The associations between MPO-DNA and the presence of VTE were explored by means of logistic regression analysis and expressed as odds ratios (OR) with corresponding 95% CIs. For these analyses, patients were stratified into two groups based on the 75th percentile of serum MPO-DNA levels, with those ≤75th percentile comprising the control group. To assess the predictive value of MPO-DNA, D-dimer, and CRP at varying cut-off values for VTE, a receiver-operating characteristic (ROC) curve was generated, and the area under the curve (AUC) was calculated. The 95% confidence intervals (95% CIs) were also calculated when appropriate. A two-sided α of <0.05 was considered statistically significant.

## Results

### Demographic and Clinical Characteristics

A total of 119 spontaneous ICH patients were screened. Two patients met exclusion criteria: one for pre-existing VTE prior to admission, and another due to length of stay <3 days. Consequently, 117 patients were included in the final cohort.

The demographic and clinical characteristics are shown in Table 1 . Among these patients, 21 (17.8%) developed VTE, while 96 (82.2%) did not. The median age of the ICH patient was 63 (54–69) years, with 72 patients (61%) being male. Patients with VTE were significantly older than those without VTE (73 [69–80] years vs. 60 [51–68] years, *P* < 0.001). Furthermore, 104 patients (88.1%) had at least one underlying comorbidity, such as hypertension, diabetes, coronary atherosclerotic heart disease, and cerebral infarction. No statistically significant differences were observed in gender or underlying comorbidities between patients with and without VTE. Due to concerns about worsening hemorrhage and initial contraindication, none of the patients received pharmacological prophylaxis. Notably, patients with VTE had a significantly longer length of hospital stay compared with patients without VTE (16 [11–23] days vs. 12 [10–15] days, *P* = 0.030).

**Table 1:** Demographic and clinical characteristics of 117 patients with ICH

	All patients ( *n* = 117)	VTE positive ( *n* = 21)	VTE negative ( *n* = 96)	*P*
Age (y), median (IQR)	62 (54–69)	72 (69–79)	60 (52–68)	<0.001
Gender				
Male	71 (60.7)	11 (52.4)	60 (62.5)	0.388
Female	46 (39.3)	10 (47.6)	36 (37.5)	
Comorbidities	103 (88.0)	17 (81.0)	86 (89.6)	0.270
Hypertension	90 (76.9)	15 (71.4)	75 (78.1)	0.509
Coronary artery disease	16 (13.6)	4 (19.0)	12 (12.5)	0.429
Diabetes mellitus	25 (21.2)	4 (19.0)	21 (21.9)	0.775
Chronic kidney disease	13 (11.0)	2 (9.5)	11 (11.5)	0.798
Pneumonia	71 (60.7)	18 (85.7)	53 (55.2)	0.010
Glasgow Coma Scale	15 (14–15)	14 (12–15)	15 (15–15)	0.033
Caprini score				0.179
0–2	0	0	0	
3–4	1	1	0	
≥5	**116**	**20**	**96**	
Length of stay (d), median (IQR)	13 (10–16)	16 (11–23)	12 (10–15)	0.034

Abbreviations: ICH, intracerebral hemorrhage; IQR, interquartile ranges; VTE, venous thromboembolism.

Note: The *P* -values indicate the differences between patients with VTE and without VTE. **Continuous variables were calculated using the Mann-Whitney U test. Categorical variables were analyzed using the Chi-square test.** *P* < 0.05 was considered statistically significant.

The values were presented in numbers (percentages), unless otherwise stated.

### MPO-DNA and Laboratory Findings

**Fig. 1:**
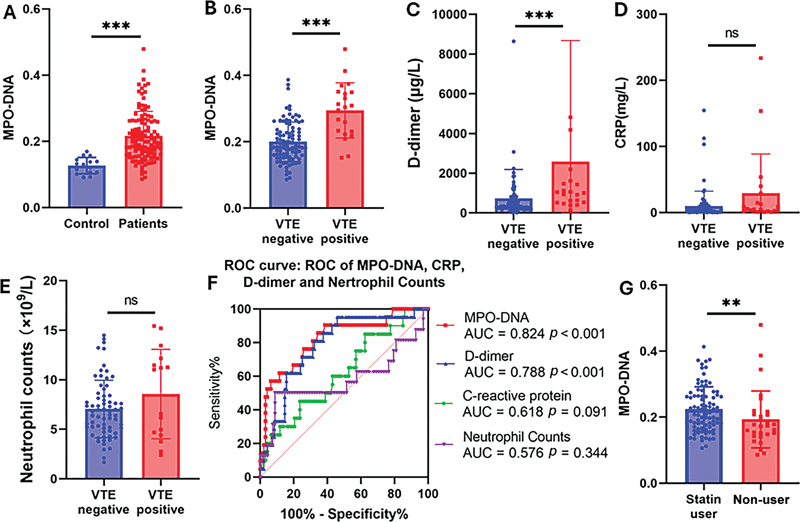
( **A** ) Serum levels of MPO-DNA in healthy control or patients with intracerebral hemorrhage (ICH). ( **B–E** ) Levels of MPO-DNA ( **B** ), D-dimer ( **C** ), C-reactive protein ( **D** ), and neutrophil counts ( **E** ) in ICH patients with or without venous thromboembolism (VTE). ( **F** ) The ROC curve analyses for MPO-DNA, C-reactive protein, D-dimer, and neutrophils. ( **G** ) Serum levels of MPO-DNA in ICH patients treated with statins or not. AUC, area under the curve; CRP, C-reactive protein; ROC, receiver operating characteristic. ** *P* = 0.007. *** *P* < 0.001.

The median MPO-DNA levels in 117 patients with ICH and 15 healthy controls were 0.204 (0.162–0.262) and 0.130 (0.106–0.142), respectively, demonstrating a statistically significant difference ( *P* < 0.001) ( Fig. 1A ).

The levels of MPO-DNA and D-dimer were significantly higher in patients with VTE compared to those without VTE ( *P* < 0.05) ( Fig. 1B, C ). However, no statistically significant difference was observed in CRP levels and neutrophil counts between the two cohorts ( Fig. 1D, E ). A detailed comparison of the data between the two groups is presented in Table 2 .

**Table 2:** Initial laboratory findings of 117 patients with ICH

	All patients ( *n* = 117)	VTE positive ( *n* = 21)	VTE negative ( *n* = 96)	*P*
C-reactive protein (mg/L)	3.97 (1.18–9.53)	5.28 (2.45–18.30)	3.95 (1.16–8.50)	0.091
Neutrophils counts (×10 ^9^ /L)	6.79 (4.90–9.23)	8.79 (4.62–11.64)	6.56 (4.55–11.36)	0.538
D-dimer (μg/L)	409 (263–842)	1,015 (535–1,471)	336 (244–594)	<0.001
MPO-DNA	0.204 (0.162–0.262)	0.304 (0.231–0.349)	0.188 (0.159–0.236)	<0.001

Abbreviations: ICH, intracerebral hemorrhage; VTE, venous thromboembolism.

Note: The *P* -values indicate the differences between patients with VTE and without VTE. **Data were analyzed using the Mann-Whitney U test.** *P* < 0.05 was considered statistically significant.

The values were presented in medians (interquartile ranges).

Logistic regression analysis revealed that serum MPO-DNA complex concentrations exceeding the 75th percentile demonstrating a 7.13-fold increased odds of VTE (95% CI 2.58–19.75; *P* < 0.001) compared to concentrations at or below this threshold. This association persisted after multivariable adjustment for clinically relevant confounders including age, sex, comorbidities, Glasgow Coma Scale scores, and intraventricular hemorrhage (adjusted OR 6.892, 95%CI 1.47–32.40; *P* = 0.015).

To evaluate the predictive accuracy of MPO-DNA, CRP, and D-dimer levels at different cut-off values for distinguishing between patients with and without VTE, an ROC curve was generated, and the AUC was calculated. Accordingly, the AUC values for MPO-DNA, CRP, and D-dimer were 0.814 (95% CI: 0.723–0.957), 0.675 (95% CI: 0.521–0.829), and 0.779 (95% CI: 0.670–0.888), respectively ( Fig. 1F ). The optimal cut-off values for these biomarkers in predicting VTE, along with their sensitivity and specificity values at these cut-offs, are presented in Table 3 .

**Table 3:** The ROC curve analyses for MPO-DNA, C-reactive protein, and D-dimer

Biomarker	AUC (95%CI)	Cut-off value	Sensitivity (%) (95%CI)	Specificity (%) (95%CI)
MPO-DNA	0.824 (0.719–0.928)	0.230	76.2 (54.9–89.4)	74.0 (64.4–81.7)
C-reactive protein (mg/L)	0.618 (0.481–0.754)	4.50	60.0 (38.7–78.1)	56.5 (46.3–66.2)
D-dimer (μg/L)	0.786 (0.683–0.888)	500	81.0 (60.0–92.3)	63.9 (54.0–72.8)

### Statins Reducing Serum MPO-DNA Levels

Given the potential of statins to inhibit NETosis, we compared the levels of NETs in patients who had been administered statins both before and after admission to those who had never received statin therapy. Of the **117** ICH patients, **30** had been treated with atorvastatin. The MPO-DNA levels were significantly lower in statin users than in non-users ( Fig. 1G ). The incidence of VTE was also lower in statin users (13.8%) compared to non-users (19.3%); however, this difference was not statistically significant ( Table 4 ). Importantly, no significant bleeding events or hematoma expansion was observed in statin users during hospitalization.

**Table 4:** The effect of statins on MPO-DNA levels and the incidence of VTE

	All patients ( *n* = 117)	Statin user ( *n* = 29)	Non-user ( *n* = 88)	*P*
MPO-DNA, median (IQR)	0.204 (0.162–0.262)	0.174 (0.145–0.213)	0.218 (0.172–0.264)	0.007
VTE positive, *n* (%)	21 (17.9)	4 (13.8)	17 (19.3)	0.501

Abbreviations: ICH, intracerebral hemorrhage; VTE, venous thromboembolism.

Note: The *P* -values represent comparisons between statin users and non-users. MPO-DNA **was compared using the Mann-Whitney U test.** VTE incidence **was analyzed with the Chi-square test.** *P* < 0.05 was considered statistically significant.

## Discussion

In this prospective observational cohort study, we investigated the predictive value of MPO-DNA for VTE in patients with ICH. MPO-DNA complexes independently predicted VTE risk (adjusted OR 4.21, 95% CI 1.89–9.34; *p* = 0.001), with a sensitivity of 76.2% and specificity of 73.2% in ROC analysis. Moreover, statin use was found to reduce serum MPO-DNA levels in patients with ICH, but its impact on VTE incidence was not statistically significant. The in-hospital incidence of any VTE in this study was 17.8% (21 out of 118 patients), and patients with VTE exhibited a significantly longer length of hospital stay compared with those without VTE. These finding were consistent with the prior studies. 16 17

Accumulating evidence indicates that elevated levels of NETs or their critical constituents, including cell-free DNA (cfDNA), MPO, CitH3, and NE, are associated with VTE. Diaz et al assessed plasma levels of cfDNA and MPO in 47 patients with DVT compared to 28 patients with a clinical suspicion of DVT but in whom DVT was excluded. Plasma DNA is elevated in patients with DVT and is associated with an increased probability of DVT. 18 Two other studies found that elevated NET formation is a hallmark of cancer-associated VTE 19 and may be a practical predictor of portal vein thrombosis in liver cirrhosis. 10 This study demonstrates a robust association between VTE and elevated serum MPO-DNA complex levels in ICH patients, which persisted after rigorous adjustment for demographic variables (age, sex), clinical severity indicators (GCS scores, intraventricular hemorrhage), and pre-existing comorbidities (adjusted OR 6.892, 95%CI 1.47–32.40; *p* = 0.015). Due to its high sensitivity and specificity at 0.230 cut-off value in distinguishing the VTE and non-VTE cases, MPO-DNA could serve as a biomarker in predicting VTE in ICH patients upon hospital admission.

Nordenholz et al tested 50 biological markers in 304 emergency department patients, finding that only D-dimer, CRP, and MPO demonstrated good diagnostic accuracy suggesting potential utility as biological marker of PE. 20 However, another study found that NET-specific markers such as H3Cit-DNA are elevated in patients with acute VTE, but the diagnostic accuracy of these markers does not exceed that of the clinically used D-dimer. 21 In our study, the MPO-DNA demonstrated excellent diagnostic accuracy on ROC analysis with high sensitivity (0.733) and specificity (0.739) at a cut-off value of 0.230 (AUC 0.840), whereas the D-dimer demonstrated higher sensitivity (0.800) but moderate specificity (0.594) at a cut-off value of 500 ng/mL (AUC 0.779). These results may be attributed to the following reasons. First, all blood samples in our study were collected upon admission, after which all enrolled patients underwent Doppler ultrasonography to exclude the possibility of VTE, making it plausible that D-dimer levels might be negative at that stage. Second, it is conceivable that some patients in the study population of Smith et al received prophylactic anticoagulation therapy, thereby preventing the onset of VTE in a subset of patients who were otherwise susceptible, ultimately leading to a reduction in the sensitivity of NETs markers in predicting VTE. 21

Intriguingly, NETs have been considered potential therapeutic targets for the prevention and treatment of patients with DVT. If the formation of NETs can be interfered without affecting normal coagulation function, this may become a more effective and safer antithrombotic strategy. 22 It was found that PAD4 knockout mice produced much less thrombi than wild-type mice after inferior vena cava stenosis. 23 Depletion of PAD4 prevented venous thrombosis in a mouse model of heparin-induced thrombocytopenia 24 and mice bearing human pancreatic tumors. 25 The use of DNase I reduced thrombosis in different mouse models of DVT. 25 26 27 Notably, medium-term treatment with DNase I prevented venous thrombosis without significant hemostatic changes in a breast cancer mice model. 28 Furthermore, several studies have showed statins reduced NETosis in patients with different diseases. 12 13 14 On the other hand, many studies have demonstrated that statins can reduce the occurrence and recurrence of VTE. 29 30 31 32 33 We aim to determine whether statin therapy can reduce the formation of NETs in patients with ICH, thereby decreasing the incidence of VTE. In our study, patients receiving statins had lower MPO-DNA levels compared to those not taking statins, suggesting that statins can inhibit NETs formation in patients with ICH. Therefore, statins may represent an effective medication for reducing NETs formation and preventing VTE in ICH patients.

Although our findings suggest that statins may reduce NETosis and potentially lower VTE risk in ICH patients, a balanced clinical assessment must consider their safety profile, particularly regarding bleeding risks. Statins are widely recognized for their cardiovascular benefits, but their effects on coagulation and intracranial hemorrhage remain debated. Concerns about statin-associated bleeding risks in ICH patients stem from earlier studies, such as the SPARCL trial, which reported a small but significant increase in hemorrhagic stroke risk among patients receiving high-dose atorvastatin (80 mg/day) for secondary prevention of ischemic stroke. 34 A meta-analysis of randomized controlled clinical trials noted that statin treatment may increase the risk of lobar cerebral microbleeds formation. 35 However, subsequent observational studies and meta-analyses have yielded conflicting results. For instance, a meta-analysis by McKinney et al involving 31 studies found no association between statin use and increased ICH incidence or hematoma expansion in ICH patients. 36 Similarly, the MUCH-Italy study observed no elevated risk of recurrent ICH with statin therapy. 37 Notably, statins may lower overall stroke recurrence in ICH patients without increasing hemorrhagic complications. 38 Importantly, abrupt discontinuation of statins in acute ICH settings may carry risks. Studies suggest that continued inpatient statin administration correlates with improved functional outcomes post-ICH, whereas statin discontinuation may exacerbate clinical outcomes. 39 40

In our cohort, none of the statin users experienced significant bleeding events or hematoma expansion during hospitalization. The lack of statistical significance in VTE incidence reduction with statins (13.3% vs. 19.5%, *P* = 0.445) may reflect sample size limitations, but importantly, no safety signals emerged. This aligns with recent guidelines suggesting that statins may be safely continued in ICH patients unless there is clear evidence of harm. 4 Nevertheless, the interplay between statins, NETosis inhibition, and coagulation requires further investigation. Statins might mitigate thrombotic risks without exacerbating hemorrhage, but larger trials are needed to confirm this hypothesis. Clinicians must weigh the potential reduction in VTE risk against theoretical bleeding concerns, individualized to patient-specific factors such as hematoma stability, comorbidities, and prior statin tolerance.

There are certain limitations in the present study. First, the direct quantification of NETs in clinical samples has not yet been standardized. Consequently, we employed an indirect approach by measuring serum MPO-DNA complex levels, which have been previously proposed as a surrogate marker for NETs formation in several clinical studies. 11 15 41 Second, our study was limited by the modest sample size of statin users. Future prospective studies with larger cohorts are warranted to further evaluate the potential of statins or other alternative agents to inhibit NETs formation and, consequently, reduce the risk of VTE.

In conclusion, our findings suggest that MPO-DNA complexes may serve as novel biomarkers for predicting VTE, while NETs represent potential therapeutic targets for VTE prevention in patients with ICH. Additionally, statins demonstrate the ability to reduce NET levels, though further validation through large-scale, multicenter randomized controlled trials (RCTs) with predefined sample sizes is imperative to confirm their efficacy and safety in VTE prevention in patients with ICH.

## Notes

**What is known about this topic?**

- Patients with spontaneous intracerebral hemorrhage (ICH) are at high risk of venous thromboembolism (VTE).
- Neutrophil extracellular traps (NETs) have been implicated in the pathogenesis of VTE.
- Statins have been shown to have anti-inflammatory and anticoagulant properties, and may affect NET formation.

**What does this paper add?**

- Serum MPO-DNA, a surrogate for NETs, is significantly associated with an increased VTE risk in ICH patients. It has a good predictive value for VTE, with an AUC of 0.824. The optimal cut-off value of 0.230 shows high sensitivity (76.2%) and specificity (74.0%), making it a potential biomarker for predicting VTE in ICH patients upon hospital admission.
- Statins can reduce serum MPO-DNA levels in ICH patients, suggesting that they can inhibit NETs formation. However, the difference in VTE incidence between statin users (13.3%) and non-users (19.5%) was not statistically significant, likely due to the small sample size. Larger-scale studies are needed to confirm the effect of statins on reducing the VTE incidence in ICH patients by inhibiting NETosis.
